# Lauroyl Arginate Ethyl Blocks the Iron Signals Necessary for *Pseudomonas aeruginosa* Biofilm Development

**DOI:** 10.3389/fmicb.2017.00970

**Published:** 2017-05-30

**Authors:** Taek-Seung Kim, So-Young Ham, Bernie B. Park, Youngjoo Byun, Hee-Deung Park

**Affiliations:** ^1^School of Civil, Environmental and Architectural Engineering, Korea UniversitySeoul, South Korea; ^2^College of Pharmacy, Korea UniversitySejong, South Korea

**Keywords:** biofilm, biofilm inhibitor, iron, lauroyl arginate ethyl, *Pseudomonas aeruginosa*

## Abstract

*Pseudomonas aeruginosa* is a ubiquitous gram-negative bacterium capable of forming a biofilm on living and non-living surfaces, which frequently leads to undesirable consequences. We found that lauroyl arginate ethyl (LAE), a synthetic non-oxidizing biocide, inhibited biofilm formation by *P. aeruginosa* at a sub-growth inhibitory concentration under both static and flow conditions. A global transcriptome analysis was conducted using a gene chip microarray to identify the genes targeted by LAE. In response to LAE treatment, *P. aeruginosa* cells up-regulated iron acquisition and signaling genes and down-regulated iron storage genes. LAE demonstrated the capacity to chelate iron in an experiment in which free LAE molecules were measured by increasing the ratio of iron to LAE. Furthermore, compared to untreated cells, *P. aeruginosa* cells treated with LAE exhibited enhanced twitching motility, a phenotype that is usually evident when the cells are starved for iron. Taken together, these results imply that LAE generated iron-limiting conditions, and in turn, blocked iron signals necessary for *P. aeruginosa* biofilm development. As destroying or blocking signals leading to biofilm development would be an efficient way to mitigate problematic biofilms, our findings suggest that LAE can aid in reducing *P. aeruginosa* biofilms for therapeutic and industrial purposes.

## Introduction

*Pseudomonas aeruginosa* is an opportunistic pathogen of various plants and animals (Stover et al., 2000; He et al., 2004). In humans, *P. aeruginosa* frequently causes serious infections in the lung airways, the urinary tract, of burns, etc., especially in immunocompromised patients (Driscoll et al., 2007). In addition, *P. aeruginosa* can contaminate medical devices such as catheters and joint prostheses, which can lead to serious medical complications (Weinstein and Darouiche, 2001). However, it is difficult to appropriately treat these infections or contamination by *P. aeruginosa* (Percival et al., 2015), mainly because *P. aeruginosa* can form a biofilm on both inert and living surfaces (Costerton et al., 1999). A biofilm is a surface-attached microbial community embedded in a self-produced hydrated polymeric matrix. Because the diffusion of antibiotics or biocides into cells across the polymeric matrix is retarded by a biofilm and biofilm cells grow slowly, biofilm cells are more resistant to antimicrobial agents than the corresponding planktonic cells (Costerton et al., 1999).

In a manner, similar to that exhibited in other bacteria, biofilm development in *P. aeruginosa* occurs in consecutive stages: initial attachment, microcolony formation, maturation into a differentiated biofilm, and dispersal of the planktonic cells. Studies over the past two decades have identified various stage-by-stage signals or cues that trigger biofilm development at the molecular level. The signal molecules leading to biofilm development that have been most frequently studied are autoinducers (e.g., 3-oxododecanoyl homoserine lactone), which are produced in response to the local population density (Davies et al., 1998). Autoinducers are essential for the differentiation of *P. aeruginosa* into a mushroom-like mature biofilm structure (Davies et al., 1998). Cyclic diguanylate (c-di-GMP) is a secondary metabolite that is synthesized intracellularly by various bacteria including *P. aeruginosa*, and it affects the various stages of *P. aeruginosa* biofilm development. High levels of cellular c-di-GMP facilitate biofilm formation, while low levels increase the dispersal of planktonic cells from a mature biofilm (Borlee et al., 2010). Intracellular iron has also been reported to serve as a signal for *P. aeruginosa* in the initial attachment (O’Toole and Kolter, 1998a), microcolony formation (Singh et al., 2002), and maturation into a differentiated biofilm (Banin et al., 2005). In addition, cis-decenoic acids, 4-quinolones, and diketopiperazines have been reported to be signal molecules for biofilm development in *P. aeruginosa* (Jimenez et al., 2012).

Destroying or blocking the signal molecules involved in biofilm development is a promising strategy for mitigating noxious *P. aeruginosa* biofilms. This approach has the advantage of reducing the appearance of resistant strains in response to antibiotic administration (Hentzer et al., 2003). Various approaches have been suggested for this strategy, such as the introduction of enzymes that degrade the autoinducers [e.g., acylase and lactonase (Dong and Zhang, 2005)], molecules that block the binding of the autoinducers to their receptors [e.g., furanone C-30 (Hentzer et al., 2002) and 6-gingerol (Kim et al., 2015)], inhibitors of the synthesis of cellular c-di-GMP [e.g., nitric oxide (Plate and Marletta, 2012) and raffinose (Kim et al., 2016)], and iron chelating molecules [e.g., lactoferrin (Costerton et al., 1999)].

**FIGURE 1:**
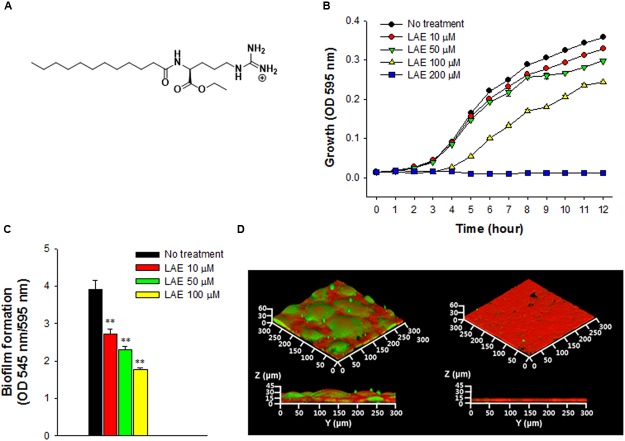
Effect of lauroyl arginate ethyl (LAE) on *Pseudomonas aeruginosa* growth and biofilm formation. **(A)** Chemical structure of LAE. **(B)** Growth at different concentrations of LAE. Growth was analyzed by hourly measurements of the OD at 595 nm. The error bars indicate the standard deviation of three measurements. **(C)** Biofilm formation in borosilicate bottles at different concentrations of LAE. Biofilm formation was analyzed by measuring the ratio of the OD at 545 nm/OD at 595 nm. The error bars indicate the standard deviation of three measurements, ^∗∗^*P* < 0.005 versus no treatment. **(D)** Confocal laser scanning microscopy (CLSM) images of 48-h biofilms formed on glass slides without (left images) and with 50 μM LAE (right images). Green and red colors in the biofilms, respectively, indicate carbohydrates and proteins which were stained by Con-A and Ruby.

Lauroyl arginate ethyl (LAE) is a broad-spectrum, synthetic, non-oxidizing biocide (Kim and Park, 2016). It has a cationic arginine moiety and a hydrophobic lauric acid tail (**Figure 1A**), which facilitates the killing of microorganisms via the lysis of the cell membrane (Rodriguez et al., 2004). In the human body, LAE can be readily hydrolyzed into nutritional components such as lauroyl arginine and arginine (Hawkins et al., 2009). Thus, LAE has been widely employed as a preservative in the cosmetics, food, and beverage industries (Kuhnert, 2002; Becerril et al., 2013). Interestingly, LAE has been shown to inhibit biofilm formation by *P. aeruginosa* when used at a sub-growth inhibitory concentration (see the “Results” section). Nevertheless, the mechanism by which LAE prevents the development of a *P. aeruginosa* biofilm under various conditions is not yet known.

The primary goal of this study was to determine the mechanism of the anti-biofilm activity of LAE. Initially, we investigated global gene expression in *P. aeruginosa* treated with and without LAE at a sub-growth inhibitory concentration. Genes associated with iron acquisition and signaling were highly activated by LAE, which suggested the hypothesis that LAE generated an iron-limiting condition. To test this hypothesis, we measured the iron chelating activity of LAE and investigated the phenotype of *P. aeruginosa* cells under iron limitation (i.e., the twitching motility). The results suggested a possible anti-biofilm mechanism for LAE.

## Materials and Methods

### Bacterial Strain and Culture Condition

*Pseudomonas aeruginosa* PA14 was used to test the effect of LAE (CDI, Hwaseong, South Korea) at a sub-growth inhibitory concentration. *P. aeruginosa* was cultured in a shaking incubator at 250 rpm and 37°C in AB medium (300 mM NaCl, 50 mM MgSO_4_, 0.2% vitamin-free casamino acids, 10 mM potassium phosphate, 1 mM L-arginine, and 1% glucose, pH 7.5) (Kim and Park, 2013). The effect of LAE on growth was evaluated by inoculating an overnight culture of *P. aeruginosa* to an optical density (OD) at 595 nm of approximately 1.2 in AB medium supplemented with different concentrations of LAE. Growth was measured by the OD at 595 nm using a UVmini-1240 spectrophotometer (Shimadzu, Kyoto, Japan).

### Static Biofilm Formation Assay

A static biofilm formation assay was used to test the effect of LAE on biofilm formation in *P. aeruginosa*. An overnight culture of *P. aeruginosa* was diluted in fresh AB medium (1:20) with and without LAE (10, 50, and 100 μM). Aliquots of 3 mL of the diluted culture were placed into borosilicate bottles and incubated at 37°C for 24 h without agitation. After 24 h incubation, the absorbance of the suspended cells in the borosilicate bottles was measured by the OD at 595 nm using a UVmini-1240 spectrophotometer (Shimadzu). After decanting the suspended cells, the biofilm cells that formed on the surface of the borosilicate bottles were stained for 30 min using crystal violet (1.0%). The stained cells were then washed with deionized (DI) water to remove the residual crystal violet. The crystal violet in the biofilm cells was then eluted in ethanol (100%). The absorbance of the eluted crystal violet was measured by the OD at 545 nm using a UVmini-1240 spectrophotometer (Shimadzu). The OD at 545 nm was normalized to the OD 595 nm to quantify the biofilm formation (O’Toole and Kolter, 1998b).

### Biofilm Formation in a Drip-Flow Reactor

Biofilm formation in a drip-flow reactor (DFR-110, BioSurface Technologies, Bozeman, MT, United States) was evaluated using a procedure from a previous study (Goeres et al., 2009). A glass slide was immersed in a solution in which an overnight culture of *P. aeruginosa* (1 mL) was diluted in fresh AB medium (19 mL) in a petri dish. The slide was incubated at 37°C for 24 h to allow a biofilm to form on the slide. The slide was inserted into a drip-flow biofilm reactor. Fresh AB medium with or without LAE (50 μM) was continuously fed into the reactor by a peristaltic pump at 18 mL/h at 37°C for 48 h. After the reactor operation was complete, the slide was washed with phosphate-buffered saline (PBS) (pH 7.2). The biofilm cells on the slide were stained with Con-A (Sigma–Aldrich, St. Louis, MO, United States) for 20 min. After washing the slide with PBS again, the biofilm cells were stained with Ruby (Invitrogen, Carlsbad, CA, United States) for 20 min. The stained biofilm cells were washed twice with DI water to remove the residual dye. Con-A and Ruby were, respectively, used to stain the carbohydrates and proteins in the biofilm. The biofilms were observed by confocal laser scanning microscopy (CLSM) (Carl Zeiss, LSM700, Jena, Germany). CLSM images were obtained using a 20x objective lens under a green fluorescence light for Con-A staining and red fluorescence light for Ruby staining. A stack of 3-D images was used to analyze the average thickness (μm) and volume (μm^3^/μm^2^) of a biofilm using the Comstat2 program from the ImageJ software (Heydorn et al., 2000; Vorregaard, 2008).

### Total RNA Extraction

To carry out a microarray analysis, the total RNA was extracted using TRIzol reagent (Invitrogen, Carlsbad, CA, United States) from planktonic *P. aeruginosa* cells treated with and without LAE following the manufacturer’s instructions. An overnight culture of *P. aeruginosa* was diluted in fresh AB medium to an OD at 595 nm = 0.3. Aliquots of 3 mL of the diluted culture were placed in borosilicate bottles with or without 50 μM LAE and further cultured at 37°C for 4 h without agitation. After centrifugation at 8,000 ×*g* at 4°C for 10 min, the culture supernatant was discarded. The *P. aeruginosa* pellets were resuspended in 1 mL of TRIzol reagent. The resuspended cells were mixed with 0.2 mL chloroform (99.9%) and centrifuged at 12,000 ×*g* at 4°C for 15 min. The aqueous phase was subsequently transferred to a 1.5 mL micro tube and 0.5 mL isopropyl alcohol (100%) was added. The mixture was centrifuged at 12,000 ×*g* at 4°C for 10 min. The pellet was washed with 1 mL ethanol (75%) and centrifuged again at 7,500 ×*g* at 4°C for 5 min. The pellet (i.e., the total RNA) was dissolved in diethyl pyrocarbonate-treated water. The concentration and purity of the total RNA were, respectively, determined by the OD at 260 nm and the 260/280 nm ratio with an Agilent Bioanalyzer 2100 (Palo Alto, CA, United States). A total RNA concentration of greater than 100 ng/μL and an OD 260/280 nm ratio of 2.0 were used for subsequent analyses.

### Microarray Analysis

Gene expression in *P. aeruginosa* cells treated with and without LAE was analyzed in duplicate by the SurePrint G3 custom gene expression array (Agilent Technology, Santa Clara, CA, United States). All of the procedures followed the manufacturer’s protocols and were conducted at Macrogen Inc. (Seoul, South Korea). RNA labeling and hybridization were performed with the Agilent one-color microarray based gene expression analysis protocol (Ver. 6.5). The total RNA of each sample (200 ng) was amplified and labeled with Cy3-dCTP. The labeled cRNAs were purified using an RNeasy Mini kit (Qiagen, Valencia, CA, United States). The concentration of the labeled cRNAs (pmol Cy3/μg cRNA) was determined using a NanoDrop ND-1000 (Wilmington, DE, United States). The labeled cRNAs (600 ng) were fragmented by the addition of 5 μL of a 10x blocking agent and 1 μL of 25x fragmentation buffer, and were then incubated at 60°C for 30 min. Finally, the labeled cRNAs were diluted with 25 μL of 2x GE hybridization buffer. The hybridization solution (50 μL) was dispensed into the gasket slide and assembled on a SurePrint G3 custom gene expression array 8 × 60K (Agilent Technology). The gasket slides were incubated for 17 h at 65°C in an Agilent hybridization oven (G2545A, Agilent Technology) and washed at room temperature using the Agilent protocol. The hybridized array was immediately scanned using an Agilent SureScan Microarray Scanner (Agilent Technologies). Array data export processing and analysis were performed using the Agilent Feature Extraction software v11.0.1.1.

### Gene Expression Omnibus (GEO) Accession Number

The microarray data were deposited at the National Center for Biotechnology Information. The GEO accession number of the microarray data is GSE96844.

### Quantitative Reverse Transcription PCR (RT-qPCR)

The expression of the genes related to the acquisition and storage of iron by *P. aeruginosa* treated with LAE was compared and quantified by RT-qPCR. The primer sets for the genes are listed in Supplementary Table S1. RT-qPCR was performed with a Bio-Rad CFX-96 system (Bio-Rad, Hercules, CA, United States) with a one-step SYBR PrimeScript RT-PCR kit (Takara Bio, Otsu, Japan). The reaction mixture for RT-qPCR consisted of 10 μL of 2x SYBR RT-PCR buffer, 1 μL of PrimeScript enzyme mix, 0.4 μL of 50x ROX Reference Dye I, 10 pmol of forward and reverse primers (1.0 μL of each solution), 100 ng/μL template RNA (1 μL), and RNase free water to a final volume of 20 μL. The thermal conditions of the reaction for each of the target genes were: cDNA synthesis at 42°C for 5 min, initial denaturation at 95°C for 10 s in step for reverse transcription, 40 denaturation cycles at 95°C for 5 s each, annealing at 60°C for 10 s, and extension at 63°C for 34 s. The fluorescence signal intensity was measured at the end of the extension step. A dissociation protocol was performed under conditions of 95°C for 15 s, 60°C for 1 min, and 95°C for 15 s for the absence of a non-specific amplicon.

### Iron Chelation Assay

Ferrous (Fe^2+^) and ferric (Fe^3+^) iron stock solutions were prepared with final concentrations from 20 μM to 20 mM by dissolving FeCl_2_ or FeCl_3_ in DI water. LAE-Fe chelation solutions were prepared by mixing 50 μL of ferrous or ferric iron solution and 1 mL of LAE solution (100 μM). The Fe/LAE molar ratios were 0, 0.1, 0.2, 0.5, 1.0, 2.0, 5.0, and 10.0. The mixed solution was gently shaken for 4 h. Chromatographic analyses were performed using an Agilent 1260 series high-performance liquid chromatograph (HPLC) (Santa Clara, CA, United States) with a reverse-phase semi-preparative column (Phenomenex Gemini-NX C18, 110 Å, 150 mm × 10 mm, 5 μm) for 40 min at a flow rate of 0.5 mL/min with a 35% isocratic acetonitrile solution in DI water. The temperature of the autosampler and the column compartment were 45°C. UV detection was carried out at a wavelength of 200 nm. The HPLC system was injected with 10 μL of each concentration of the mixed solution at least three times. The LAE peaks with a retention time of 17.7 min were used for the quantification analysis (Pezo et al., 2012).

### Twitching Motility Assay

The twitching motility of the *P. aeruginosa* cells was assayed using the procedure of Singh et al. (2002). An overnight culture of *P. aeruginosa* was diluted in fresh AB medium to an OD at 595 nm of approximately 0.1 and supplemented with LAE. The diluted solution (2 μL) was stab-inoculated on the bottom of a 1% AB agar plate, which was incubated at room temperature for 2 days. The zone of twitching was measured with a Vernier caliper (Mitutoyo, Tsukuba, Japan).

## Results

### Inhibition of Biofilm Formation under Sub-growth Inhibitory Concentration at a LAE

The growth of *P. aeruginosa* was evaluated at different concentrations of LAE (0, 10, 50, 100, and 200 μM) by measuring the OD at 595 nm for 12 h (**Figure 1B**). Growth decreased as the LAE concentration increased. *P. aeruginosa* showed relatively less growth inhibition at 10 and 50 μM than at 100 μM, especially during the early period (<5 h). *P. aeruginosa* could not grow at 200 μM. *P. aeruginosa* biofilm formation was investigated in borosilicate bottles under static conditions under the sub-growth inhibitory conditions at LAE (10, 50, and 100 μM). **Figure 1C** shows that biofilm formation (545 nm/595 nm) was reduced by 30.3–54.7% as the LAE concentration increased. Inhibition of biofilm formation was further verified by evaluating the biofilm development on a glass surface under flow conditions. **Figure 1D** shows CLSM images of 48-h biofilms in the presence and absence of 50 μM LAE. The biofilm in the absence of LAE had a characteristic thick, mushroom-like structure (average thickness = 33.8 μm, average volume = 17.5 μm^3^/μm^2^), while the biofilm in the presence of LAE had a thin, flat structure (average thickness = 12.4 μm, average volume = 8.0 μm^3^/μm^2^). Furthermore, the biofilm in the absence of LAE had more carbohydrates than the biofilm in the presence of LAE (i.e., there was an increase in the green color in the CLSM image of the biofilm in the absence of LAE). The decreased production of carbohydrates in the biofilm in the presence of LAE was verified by directly measuring the total carbohydrates in the biofilm matrix (**Supplementary Figure S1**).

### Analyses of Global Transcriptome at a Sub-growth Inhibitory Condition

**FIGURE 2:**
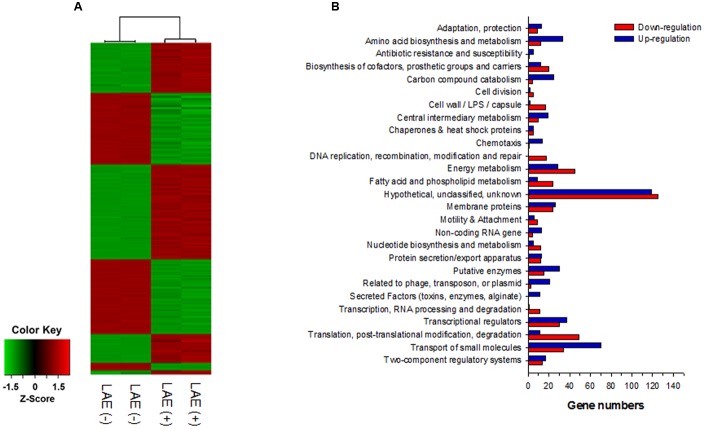
Global transcriptome analysis for *P. aeruginosa* with and without 50 mM LEA. **(A)** Heatmap of the expression of 4,922 genes with hierarchical clustering. **(B)** Classification of up- and down-regulated genes based on metabolic function.

A global transcriptome analysis based on a microarray was conducted to identify the *P. aeruginosa* genes targeted by LAE at a sub-growth inhibitory concentration (50 μM). After a quality check of the microarray data, 4,922 of 6,443 genes were selected for comparison of *P. aeruginosa* with and without LAE. **Figure 2A** is a heat map with hierarchical clustering that demonstrates that the relative gene expression between the two groups was significantly different, but did not differ within groups. A total of 1,061 of 4,922 genes were differentially expressed in the two groups with a twofold cut-off: 548 and 513 genes were, respectively, up-or down-regulated (Supplementary Table S2). Classification of the differentially expressed genes according to metabolic functions (Stover et al., 2000) (**Figure 2B**) showed that diverse metabolic categories were affected by LAE, although the genes in hypothetical and unknown categories were the most abundant. If a fivefold change was used instead, 44 and 22 genes were, respectively, up-or down-regulated (Supplementary Table S3). Interestingly, many genes associated with cellular iron acquisition, signaling, and storage were found on the list. For example, the genes responsible for pyoverdine biosynthesis and transport (*pvd* and *fpv* genes), and pyochelin biosynthesis and transport (*pch* and *fpt* genes) were up-regulated, while the activity of bacterioferritin biosynthesis genes (*bfr*) was down-regulated. The fold-changes in expressions of the pyochelin-, pyoverdine-, and bacterioferritin-associated genes are summarized in **Table 1**.

**Table 1:** Fold-changes in expression of the pyochelin, pyoverdine, and bacterioferritin genes in response to 50 mM lauroyl arginate ethyl (LAE).

ID	Gene name	Gene product	Fold-change
Pyochelin system			
PA14_09340	*fptA*	Fe(III)-pyochelin outer membrane receptor	4.8
PA14_09350	*fptB*	Hypothetical protein	4.9
PA14_09380	*fptX*	Probable transporter	7.1
PA14_09210	*pchA*	Salicylate biosynthesis isochorismate synthase	6.4
PA14_09220	*pchB*	Salicylate biosynthesis protein PchB	7.8
PA14_09230	*pchC*	Pyochelin biosynthetic protein PchC	5.4
PA14_09240	*pchD*	Pyochelin biosynthetic protein PchD	6.7
PA14_09260	*pchR*	Transcriptional regulator PchR	4.7
PA14_09270	*pchE*	Dihydroaeruginoic acid synthetase	5.4
PA14_09280	*pchF*	Pyochelin synthetase	7.6
PA14_09290	*pchG*	Pyochelin biosynthetic protein PchG	5.5
PA14_09300	*pchH*	Probable ATP-binding component of ABC transporter	4.0
PA14_09320	*pchI*	Probable ATP-binding component of ABC transporter	4.4
Pyoverdine system			
PA14_33680	*fpvA*	Ferripyoverdine receptor	3.6
PA14_09970	*fpvB*	Second ferric pyoverdine receptor FpvB	1.6
PA14_33810	*pvdA*	l-Ornithine N5-oxygenase	5.3
PA14_33650	*pvdD*	Pyoverdine synthetase D	2.2
PA14_33690	*pvdE*	Pyoverdine biosynthesis protein PvdE	1.7
PA14_33700	*pvdF*	Pyoverdine synthetase F	1.9
PA14_33270	*pvdG*	Protein PvdG	2.6
PA14_33500	*pvdH*	l-2,4-diaminobutyrate:2-ketoglutarate 4-aminotransferase, PvdH	5.6
PA14_33610	*pvdI*	Probable non-ribosomal peptide synthetase	4.4
PA14_33630	*pvdJ*	Protein PvdJ	2.2
PA14_33280	*pvdL*	Peptide synthase PvdL	3.3
PA14_33720	*pvdN*	Protein PvdN	2.5
PA14_33710	*pvdO*	Protein PvdO	6.5
PA14_33740	*pvdP*	Protein PvdP	2.7
PA14_33820	*pvdQ*	3-Oxo-C12-homoserine lactone acylase PvdQ	4.1
PA14_33260	*pvdS*	Sigma factor PvdS	2.9
PA14_39800		ECF sigma factor, FemI	3.3
Other iron acquisition genes			
PA14_09160	*bfrA*	Bacterioferritin	-15.1
PA14_18670	*bfrB*	Bacterioferritin	-33.5
PA14_72970	*tonB*	TonB protein	8.9

**FIGURE 3:**
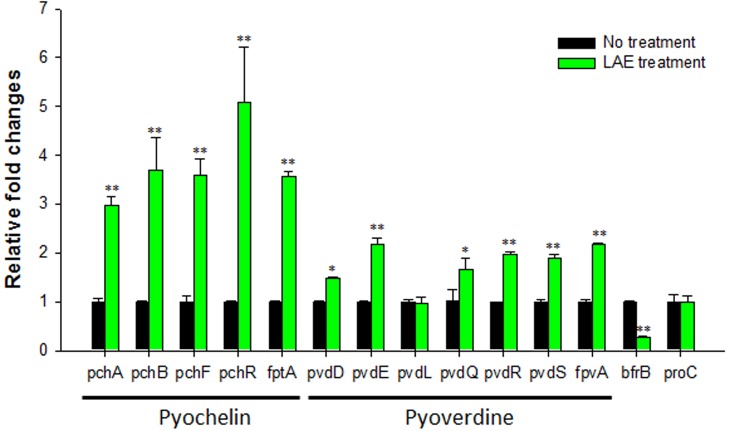
Effect of LAE on the expression of iron acquisition, signaling, and storage genes in *P. aeruginosa* with and without 50 mM LEA. The fold-change was defined as the relative copy number of the cDNA of each gene normalized by the copy number of the cDNA of the corresponding gene in the absence of LAE. The error bars indicate the standard deviation of three measurements. ^∗^*P* < 0.05 versus no treatment, ^∗∗^*P* < 0.005 versus no treatment.

To verify the expression of the iron acquisition, signaling, and storage genes, RT-qPCR was conducted for representative pyochelin, pyoverdine, and bacterioferritin genes (*pchA*, *pchB*, *pchF*, *pchR*, *fptA*, *pvdD*, *pvdE*, *pvdL*, *pvdQ*, *pvdR*, *pvdS*, *fpvA*, and *bfrB*), as shown in **Figure 3**. The results concur with the microarray results: the pyochelin and pyoverdine-associated genes were up-regulated (1.5- to 5.1-folds), except for *pvdL* (0.98-fold), and a bacterioferritin gene (*bfrB*) was down-regulated (0.3-fold). The expression of the *proC* housekeeping gene was not affected by LAE (*P* > 0.05). A noteworthy result was the higher activation of the pch genes than the pvd genes (*P* < 0.05).

### Iron Chelation Activity by LAE

**FIGURE 4:**
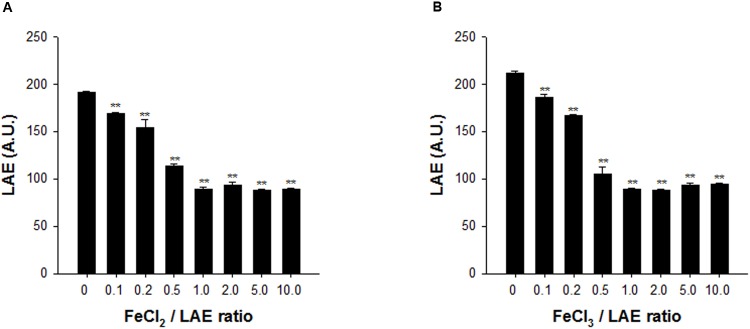
Analysis of iron chelation by LAE at different ratios of iron and LAE. The LAE concentration was fixed at 100 mM. **(A)** Ferrous iron (Fe^2+^) chelation by LAE. **(B)** Ferric iron (Fe^3+^) chelation by LAE. The error bars indicate the standard deviation of three measurements. ^∗∗^*P* < 0.005 versus no treatment of ferric or ferrous iron.

The iron chelation activity of LAE was measured by HPLC, by determining the unbound LAE as the ferrous or ferric iron concentration was increased (the ratio of the iron to the LAE concentration was varied between 0 and 10). **Figure 4** shows that the LAE signals in the HPLC chromatograms decreased asymptotically with an increase in the iron concentration when the LAE concentration was fixed at 100 μM, demonstrating that LAE could chelate iron. However, LAE appeared to be a weak iron chelator because he HPLC analysis indicated that the LAE signal did not show a further decrease, even when the ratio was as high as 10 (i.e., there was a 53.7 and 55.5% reduction in ferrous and ferric iron, respectively, at a ratio of 10). Another characteristic of LAE is its specificity. LAE had chelating activity for both ferric iron and ferrous iron. We also noted that the putative genes for ferrous iron uptake (*feo*) (Cartron et al., 2006) were up-regulated in response to LAE treatment in the microarray analysis (Supplementary Tables S2, S3).

### Effect of LAE on Twitching Motility

**FIGURE 5:**
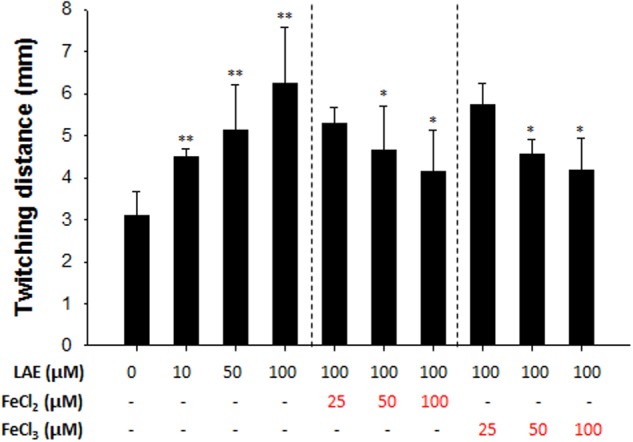
Effect of LAE on *P. aeruginosa* twitching motility. Twitching distances were estimated by measuring the radius of the cell clusters on plates. The error bars indicate the standard deviation of three measurements. ^∗^*P* < 0.05 versus 100 mM LAE treatment, ^∗∗^*P* < 0.005 versus no treatment.

Twitching motility is a translocation mode of *P. aeruginosa*, and it involves type IV pili which can attach to a surface and pull *P. aeruginosa* in a forward direction (Burrows, 2012). **Figure 5** shows that LAE stimulated the twitching motility by 146–203% at the agar and plastic interfaces in a concentration-dependent manner (10–100 μM). On the other hand, the twitching motility was gradually decreased by an increase in the supplemental ferrous or ferric iron concentration.

## Discussion

**FIGURE 6:**
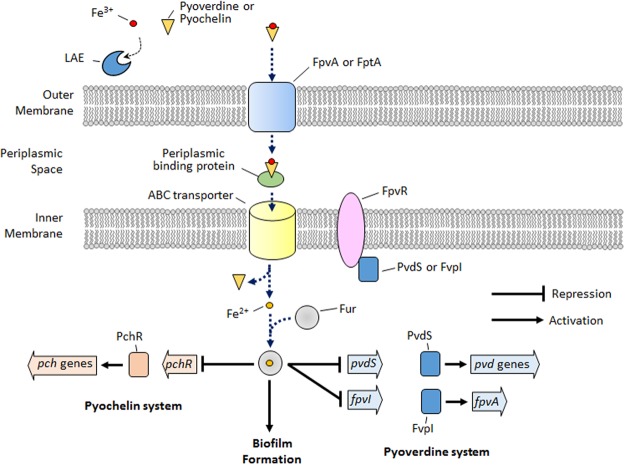
Schematic depicting the simplified iron signaling for the pyoverdine/pyochelin system and biofilm formation in *P. aeruginosa*. LAE competes with pyoverdine or pyochelin to chelate extracellular iron. Upon iron starvation via iron chelation by LAE, *P. aeruginosa* activates the genes for the pyoverdine and pyochelin systems and inhibits biofilm formation, which is the opposite condition to that depicted in the schematic.

Iron is essential for bacterial growth, primarily because it acts as a cofactor for enzymes related to oxygen metabolism, electron transport, and nucleic acid synthesis (Braud et al., 2009). However, iron is toxic to bacteria at higher concentrations due to the formation of reactive oxygen species (Cornelis and Andrews, 2012). Therefore, it is important for the bacteria to maintain an optimal intracellular iron concentration. Similar to the situation in many other bacteria, iron homeostasis in *P. aeruginosa* is regulated by the Fur protein (Cornelis et al., 2009). Extracellular iron cannot be transported into the cytoplasm via passive transport (Banin et al., 2005). Therefore, many bacteria use endogenous siderophores to transport extracellular iron. Pyochelin and pyoverdine are representative ferric iron chelating molecules (i.e., siderophores) for intracellular iron acquisition, and are secreted by certain pseudomonads including *P. aeruginosa* (Banin et al., 2005). Pyoverdine is reported to have a higher affinity for ferric iron than pyochelin (Banin et al., 2005). **Figure 6** shows that, for ferric iron transport into the cytoplasm, the ferric iron initially binds to extracellularly secreted pyoverdine or pyochelin, and the bound ferric iron is transported across the outer membrane via an energy-coupled TonB-dependent receptor (i.e., FpvA for pyoverdine and FptA for pyochelin). After binding to a periplasmic binding protein in the periplasmic space, the bound ferric iron is transported across the inner membrane via an ATP-binding cassette transporter (ABC) transporter, while the ferric ion separates from the pyoverdine or pyochelin and is reduced to ferrous iron. In the cytoplasm, ferrous iron is used for synthesis of enzymes or for storage. Excess ferrous iron is bound to a Fur protein and regulates the genes involved in iron acquisition (e.g., the pyoverdine and pyochelin systems), biofilm formation, synthesis of small RNAs, etc., directly or via extracytoplasmic sigma factors (ECF σ factors) or other regulators (Cornelis et al., 2009). For example, the Fur protein, when bound to ferrous iron, represses the genes for the ECF σ factors involved in the pyoverdine system (e.g., *pvdS* and *fpvI*) and represses genes for the transcriptional regulators of the pyochelin system (e.g., *pchR*). The genes for pyoverdine synthesis and uptake are additionally regulated by the ECF σ factors (PvdS and FpvI) that are bound to an anti-sigma factor (FpvR) (Edgar et al., 2014). Upon the binding of ferric iron to FpvA, a signal is transmitted to FpvR, which enables the FpvR to release PvdS or FpvI, and the released PvdS or FvpI, respectively, activates the *pvd* genes or the *fpvA* gene.

In this study, the microarray and RT-qPCR results demonstrated that the genes associated with iron acquisition, signaling, and storage are targeted by LAE at the sub-growth inhibitory concentration. Furthermore, an experiment that measured free LAE molecules by increasing the ratio of iron to LAE demonstrated that LAE chelates iron, which led to the hypothesis that LAE generated iron-limiting conditions. To test this hypothesis, we evaluated the twitching motility of *P. aeruginosa*. Singh et al. (2002) reported that iron-limiting conditions generated by the presence of the iron chelating molecule deferoxamine showed increased twitching motility in *P. aeruginosa*. Therefore, we conducted an experiment to test whether exposure to LAE increases the twitching motility, which showed that LAE stimulated twitching motility in proportion to the supplemental ferrous or ferric iron present. Furthermore, the twitching motility gradually decreased as the supplemental iron concentration increased. These results suggest that LAE created iron-limiting conditions, and the iron-limiting conditions were relieved by the supplemental iron.

It is reasonable to speculate that the iron-limiting conditions led to the activation of the genes associated with the pyoverdine and pyochelin systems, allowing better uptake of extracellular iron. Iron-limiting conditions generated by the LAE treatment would hinder the transport of ferric iron into the cytoplasm in *P. aeruginosa*, which in turn would cause the ferrous iron to become unbound from the Fur protein. The native Fur protein would relieve the repression of the genes for the pyoverdine and pyochelin systems (e.g., *pvdS*, *fpvI*, and *pchR*). Interestingly, in our study, the pyochelin-associated genes were more highly activated than the pyoverdine-associated genes (**Figure 3**). Two previous studies on global gene expression in *P. aeruginosa* in response to iron starvation (Ochsner et al., 2002; Palma et al., 2003) showed the same trend. These results were contrary to our expectations, because a high-affinity siderophore (i.e., pyoverdine) would be expected to show higher expression in order to bind the limited extracellular iron, as opposed to an increase in the expression of a low-affinity siderophore (i.e., pyochelin). The higher expression of the pyochelin-associated genes suggests that pyochelin may have other functions besides iron uptake. Several studies support this speculation. Pyochelin has been reported to remove iron bound to transferrin, a serum glycoprotein, to secure iron (Sriyosachati and Cox, 1986). This may contribute to the growth and virulence of *P. aeruginosa* (Cox, 1982). In addition, pyochelin catalyzes the formation of free radicals that can damage host tissues (Britigan et al., 1994).

In *P. aeruginosa*, iron acts as a signal for biofilm development as well as a micronutrient for growth. If *P. aeruginosa* cells are under iron-limiting conditions or cannot acquire iron, they cannot form a mature biofilm. Singh et al. (2002) demonstrated that biofilm formation in *P. aeruginosa* was prevented by lactoferrin, a component of human mucosal secretion that chelates iron. We also found that LAE chelates ferric and ferrous iron, and that *P. aeruginosa* treated with LAE forms thin biofilms, similar to the results of a study by Singh et al. (2002). On the other hand, Banin et al. (2005) found that mutants that could not produce pyoverdine or pyochelin could not form a mature biofilm. In addition, they demonstrated that a Fur mutant could form a mushroom-like biofilm even under iron-limiting conditions. From their results, they claimed that intracellular iron is essential for the development of a mature biofilm, and that iron is a signal for biofilm development that acts via the Fur regulator. In this regard, our results suggest that LAE can be an effective inhibitor of iron signaling for biofilm development, possibly via the Fur regulator.

Lauroyl arginate ethyl was expected to possess iron chelating activity because it contains donor groups found in common ferric (e.g., deferoxamine and deferiprone) and ferrous (e.g., ferrozine and phenanthroline) iron chelators (Haas and Franz, 2009). **Figure 1A** shows that LAE contains two types of donor groups that can potentially form ferric and ferrous iron chelate complexes: the two carbonyl groups (ester and amide) with a 1,4-relationship and a guanidine group. On the other hand, since ferric iron is a harder acid than ferrous iron, the harder ligands (e.g., hydroxamate, phenolate, and catecholate) with electronegative oxygen donor groups would preferentially coordinate with ferric iron rather than with ferrous iron (Martell et al., 1996). However, the softer ligands (e.g., ethylenediaminetetraacetic acid, trimethylenedinitrilotetraacetic acid) with both oxygen and nitrogen donor groups increase the stability of ferrous chelate, although they still have a slight preference for ferric iron. Because LAE possesses both oxygen donor groups (i.e., ester and amide groups) and a nitrogen donor group (i.e., guanidine), it might act as a softer ligand, thus increasing the relative stability of the ferrous-LAE complex.

The inhibition of biofilm formation in *P. aeruginosa* by LAE has important implications in medicine and industry. Colonization by *P. aeruginosa* in the form of biofilms on various surfaces (e.g., lung, catheters, water filters, etc.) frequently causes serious problems. However, it is difficult to eliminate *P. aeruginosa* biofilms due to their resistance to antibiotics and biocides. Our finding suggests that LAE can mitigate such problems by retarding biofilm formation. In line with reports on the vulnerability of biofilm cells to antibiotics in the presence of a biofilm inhibitor (Brackman et al., 2011), LAE might also be used to kill biofilm cells by administering the LAE along with antibiotics. In addition, the low toxicity of LAE (Ruckman et al., 2004) is an advantage in medical applications.

## Conclusion

In this study, we found that LAE, a synthetic biocide, inhibited biofilm development in *P. aeruginosa* at sub-growth inhibitory concentrations. LAE activated the genes involved in iron acquisition (e.g., the pyoverdine and pyochelin related genes) and increased twitching motility, due to the low availability of iron to *P. aeruginosa* because LAE chelated the iron. We can infer that low levels of cellular iron blocked the iron signal that led to biofilm formation. We expect that LAE can aid in the treatment of *P. aeruginosa* biofilms in medical and industrial settings as an effective biofilm inhibitor.

## Author Contributions

H-DP and YB conceived and designed the experiments; T-SK, S-YH, and BP performed the experiments; H-DP, YB, and T-SK analyzed the data; H-DP, T-SK, and YB wrote the paper; all authors approved the final version of the manuscript.

## Conflict of Interest Statement

The authors declare that the research was conducted in the absence of any commercial or financial relationships that could be construed as a potential conflict of interest.

## Supplementary Material

FIGURE S1 Total proteins and total carbohydrates in biofilms with and without LAE. ^∗^*P* < 0.05 versus no treatment.
