# Supplementary figures and images for: Lauroyl Arginate Ethyl Blocks the Iron Signals Necessary for Pseudomonas aeruginosa Biofilm Development
Source: Front Microbiol. 2017 May 30;8:970. doi: 10.3389/fmicb.2017.00970 (PMC5447684; doi:10.3389/fmicb.2017.00970)

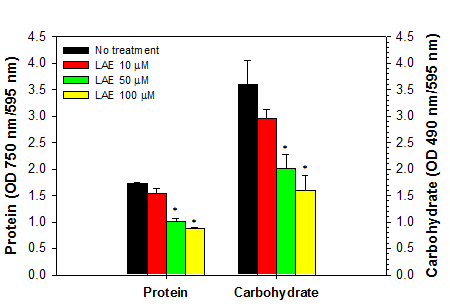

Supplement: FIGURE S1 — Total proteins and total carbohydrates in biofilms with and without LAE. ∗P < 0.05 versus no treatment. [file Image_1.TIF]
